# Supplementary material for: Cognitive and Adaptive Characterization of Children and Adolescents with KBG Syndrome: An Explorative Study
Source: J Clin Med. 2021 Apr 6;10(7):1523. doi: 10.3390/jcm10071523 (PMC8038739; doi:10.3390/jcm10071523)
Supplement: Supplementary file 1 [file jcm-10-01523-s001.pdf]

Table S1. Prevalence of uncommon clinical features in the present series of KBG syndrome patients.

**Clinical features**	**P1**	**P2**	**P3**	**P4**	**P5**	**P6**	**P7**	**P8**	**P9**	**P10**	**P11**	**P12**	**P13**	**P14**	**P15**	**P16**	**P17**	**P18**	**P19**	**P20**	**P21**	**P22**	**P23**	**P24**
Sex	M	F	M	M	F	M	M	F	F	M	F	F	M	M	M	F	M	F	F	M	M	M	F	M
**Neuropsychological anomalies**																								
Epilepsy	-	-	-	+	-	+	-	-	-	+	+	-	-	-	+	-	-	+	-	+	-	-	-	-
Motor tics	-	-	-	-	-	-	-	-	-	-	-	-	-	+	-	-	-	-	-	-	-	-	-	-
Cerebral malformation	-	n.e.	n.e.	-	+	-	-	n.e.	n.e.	-	-	n.e.	+	-	n.e.	-	n.e.	n.e.	n.e.	n.e.	-	-	+	-
**Ocular anomalies**																								
Strabismus	+	-	-	-	-	+	-	-	-	-	-	-	+	+	-	+	-	-	-	-	-	-	-	-
**Otorhinolaringological anomalies**																								
Hearing loss	-	-	-	-	-	-	-	-	-	-	-	-	+	-	-	-	-	-	+	-	-	+	-	-
Sensorineural	-	-	-	-	-	-	-	-	-	-	-	-	-	-	-	-	-	-	-	-	-	-	-	-
Conductive	-	-	-	-	-	-	-	-	-	-	-	-	+	-	-	-	-	-	+	-	-	+	-	-
**Gastroenterological problems**																								
Gastroesophageal reflux	+	-	-	+	-	-	-	-	-	-	+	-	+	+	-	+	-	-	-	-	-	-	+	-
**Treatments medication at the time of assessment**																								
	-	-	-	+<br>levetiracetam	-	+ *<br>risperdal<br>venlafaxina<br>valproic<br>acid	-	-	-	+<br>levetiracetam	+<br>carbamazepine	-	-	-	+<br>valproic<br>acid	-	-	-	-	-	-	-	-	-

P, patient; F, female, M, male; n.e., not evaluable, + positive, - negative, *P6 takes drugs because he also had a behavioural disorder
